# Analyzing Breathing Signals and Swallow Sequence Locality for Solid Food Intake Monitoring

**DOI:** 10.1007/s40846-016-0181-5

**Published:** 2016-12-09

**Authors:** Bo Dong, Subir Biswas

**Affiliations:** Department of Electrical and Computer Engineering, Michigan State University, East Lansing, MI 48824 USA

**Keywords:** Wearable sensors, Swallow detection, Food intake monitoring, Support vector machine (SVM), Hidden Markov model

## Abstract

Self-reported questionnaires are widely used by researchers for analyzing the dietary behavior of overweight and obese individuals. It has been established that questionnaire-based data collection often suffers from high errors due to its reporting subjectivity. Automatic swallow detection, as an alternative to questionnaires, is proposed in this paper to avoid such subjectivity. Existing approaches for swallow detection include the use of surface electromyography and sound to detect individual swallowing events. Many of these methods are generally too complicated and cumbersome for daily usage in a free-living setting. This paper presents a wearable solid food intake monitoring system that analyzes human breathing signals and swallow sequence locality. Food intake is identified by detecting swallow events. The system works based on a key observation that the otherwise continuous breathing process is interrupted by a short apnea during swallowing. A support vector machine (SVM) is first used for detecting such apneas in breathing signals collected from a wearable chest belt. The resulting swallow detection is then refined using a hidden Markov model (HMM)-based mechanism that leverages the known temporal locality in the sequence of human swallows. Temporal locality is based on the fact that people usually do not swallow in consecutive breathing cycles. The HMM model is used to model such temporal locality in order to refine the SVM results. Experiments were carried out on six healthy subjects wearing the proposed system. The proposed SVM method achieved up to 61% precision and 91% recall on average. Utilization of HMM in addition to SVM improved the overall performance to up to 75% precision and 86% recall.

## Introduction

According to data from the World Health Organization, worldwide obesity has increased by over 200% since 1980 [1]. It has been proven that obesity can cause coronary heart disease, type-2 diabetes, and various types of cancer [2]. Diet control and physical exercise are the two most important components of obesity control. Self-reported questionnaires are widely used by researchers for estimating both food intake and physical activity levels for high-risk individuals. In recent years, accelerometers, gyroscopes, and pressure sensors have been widely utilized for instrumented physical activity monitoring with high detection accuracy [3]. However, not many efforts on instrumented diet monitoring have been reported in the literature. Diet monitoring can reduce the subjectivity [4] associated with questionnaire-based self-reporting systems.

An instrumented system can potentially detect each instance of food/drink intake, and can have a significant impact on obesity and overall health monitoring and management. Together with the self-reporting of dietary habits at a high level, the system can quantify calorie intake trends and estimates for its users.

Non-invasive methods use biological signals such as electromyography (EMG), sound, and movement to detect swallows. Surface EMG (sEMG) and sound signals have been used to detect the activation of muscles and the sound associated with swallow events [5]. sEMG electrodes are normally attached to the bare skin in the neck region, which may cause user acceptability concerns for prolonged usage due to cosmetic and safety reasons. A two-microphone system was developed [6] for recording chewing and swallowing sound through the ear canal as well as externally through the air. In another study [7], an inertial measurement unit (with an accelerometer, a gyroscope, and a magnetometer) was attached to the chin to measure jaw movement during free chewing. Similarly, Imtiaz et al. [8] put two inertial measurement units on the shoulder and the back of the head to measure the head movement angle and an EMG unit under the chin to measure chewing activity. Microphones have been placed in the neck area near the laryngopharynx for detecting the sound of swallowing by either a stretchy band or elastic structure [9, 10]. Wang et al. [11] used a piezoelectric sensor mounted on the neck region to determine the best place for swallow detection. In one study [12], an electroglottograph sensor and a microphone were integrated into an elastic collar band tied to the neck. It has been shown that above-mentioned methods [5–12] can provide promising results. However, placing sensors in face and neck regions has cosmetic and usability issues, and thus their suitability for prolonged usage is questionable In a study [13] where a microphone and elastic bands were used, subjects consistently stated that equipment on the neck was uncomfortable and that it often impacted their swallowing habits. Respiratory inductance plethysmography (RIP) has been used for swallow detection by measuring the airflow in the trachea [14]. The RIP signal acquisition equipment used for this method is too involved to be useable for prolonged use in daily life settings. The RIP experiments [14] were conducted in a strictly controlled environment. Adib et al. [15] developed a wireless breath monitoring system using 5.46–7.25 GHz radio-frequency signals to detect minor movements of the chest caused by breathing and heartbeats. Such a system can be used without physical contact and can be used for multiple users, but it requires a specific infrastructure setup.

The idea of swallow detection through breathing signals proposed in this paper is based on the concept of swallow apnea. Anatomically, breathing is inhibited during part of the swallow process, thus causing swallow apnea. The swallowing process is divided into three steps [5]: (1) the oral preparation phase, (2) the pharyngeal phase, and (3) the esophageal phase. During the oral phase, food is chewed into a viscous bolus. The volume and viscosity of the bolus is also sensed in this phase, so that the swallowing apparatus can adapt to the bolus. In the pharyngeal phase, the bolus travels through the pharynx and passes the upper esophageal sphincter. A set of muscles is activated to propel the bolus and the epiglottis moves downward to cover the vocal folds and to protect the trachea from contamination. Finally, the bolus is pushed towards the stomach during the esophageal phase. During the pharyngeal phase, since the trachea is blocked by the epiglottis, breathing is temporarily stopped, causing apnea.

We present a wearable sensor system for solid food intake monitoring based on swallows detected in breathing signals. We detect swallows by detecting apneas extracted from breathing signals captured by a chest belt. Since the belt can be worn inside, outside, or between garments (it does not need skin contact), it has potential for prolonged comfortable daily usage without raising any cosmetic and discomfort concerns.

This paper specifically focuses on solid food intake monitoring. Future work will attempt to distinguish liquid and solid intake. We have chosen to focus on solid intake detection first because people are more likely to eat solid food over an extended period of time during a meal. Drinking, on the other hand, usually happens with one or two gulps at a time during relatively shorter periods. Also, the majority of swallows during a food intake episode consist of solid intake swallows.

It should also be noted that although the proposed system cannot detect the consumed food content and its exact associated calorie intake, it can tell when swallows happen and how long a sequence of swallows lasts. By analyzing such sequences, the estimated timing and duration of a subject’s dietary habits can be determined. For example, it can tell whether a subject skips breakfast frequently or whether they have a considerable amount of late-night snacks, which have been proven to be strongly correlated with obesity. More specifically, previous studies [16, 17] have shown that regular breakfast consumption can greatly reduce the risk of being obese or overweight. Other studies [18, 19] have demonstrated that large amounts of late-night snacks can lead to severe obesity. The recording of swallow sequences can also help detect any change in the trends of dietary habits in terms of timing, frequency, and duration of meals. Analyzing dietary habit change trends can be useful for evaluating the efficacy of various obesity management programs.

This study focuses on solid intake detection using a two-stage support vector machine-hidden Markov model (SVM–HMM) processing strategy. After the swallow sequence is recorded, an SVM is used for detecting apneas in breathing signals collected from a wearable chest belt. The resulting swallow detection is then refined using an HMM-based mechanism that leverages the known locality in the sequence of human swallows (utilizing prior knowledge of the swallowing pattern). As people usually do not swallow in consecutive breathing cycles (BCs), we are able to improve swallow detection accuracy.

The contributions of this paper are: (1) the use of a piezoelectric wearable chest belt as a non-invasive sensor for swallow detection, (2) development of a wireless data collection system for day-to-day use, (3) the use of a combination of SVM and HMM methods for processing breathing signals for solid food intake detection, and (4) experimental demonstration of the detection accuracy and effectiveness of the proposed system and the signal processing methods.

It should be noted that the presented system design was not optimized for its ergonomics, and that the system can be improved in terms of power consumption. At this stage, the system is mainly a proof-of-concept. In its product form, the system can be simplified and integrated within the chest belt, and possibly be embedded into clothes and connected to a smartwatch.

## Methods

### Sensing System Components

**Fig. 1:**
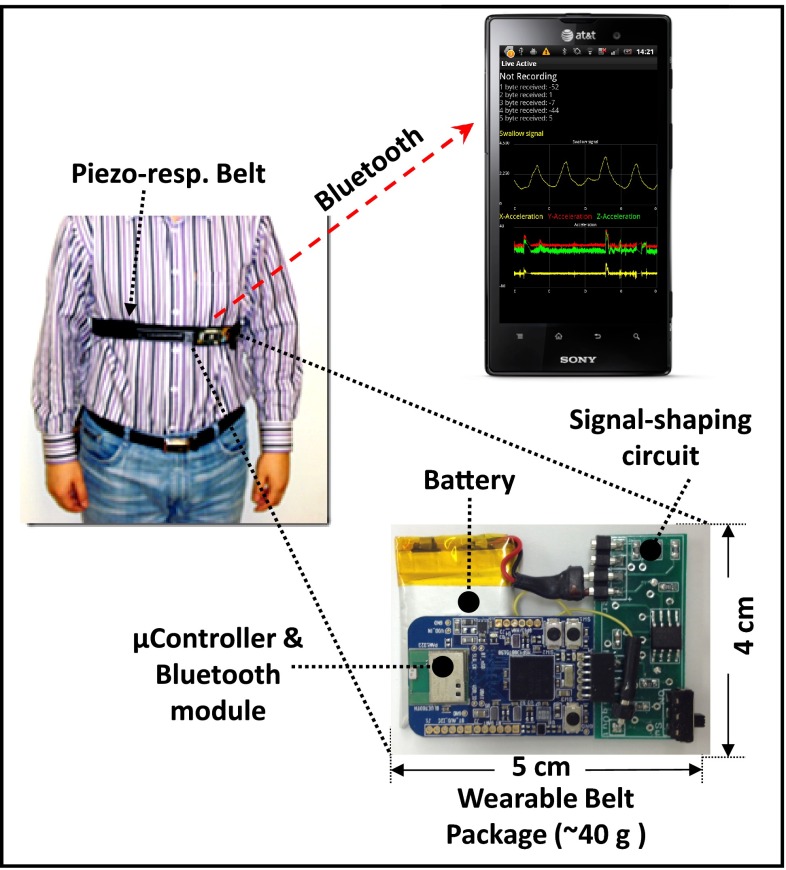
Proposed wearable wireless food intake monitoring system

As shown in Fig. 1, an embedded wearable sensor system is worn on the chest for collecting breathing signals and transmitting them to a smartphone through Bluetooth. The embedded belt system contains: (1) a piezo-respiratory belt for converting the changes of tension during breathing into a voltage signal, (2) a 20-dB amplifier and an anti-aliasing low-pass filter with a cut-off frequency at 30 Hz for maximizing the signal-to-noise ratio (SNR) and for avoiding aliasing components being sampled by the analog-to-digital converter (ADC), (3) a processor and radio subsystem (EZ430-RF256x, Texas Instruments, Texas), and (4) a 3.7-V 300-mAh polymer rechargeable battery. The entire package weighs approximately 40 g. The 300-mAh polymer battery can support the system for more than 30 h of continuous operation on a single charge. Noise control was implemented in every stage of data acquisition. Thick wires and a low-noise op-amp were adopted in the hardware design. The anti-aliasing low-pass filter and digital low-pass filter after the ADC were used to minimize the noise caused by the ADC. The amplifier gain was set to maximize the SNR. After the signal is received by the smartphone, it is stored on an SD card attached to the phone. For the 12-bit ADC and 100-Hz sampling frequency used in this paper, a 32-MB SD card can store up to 46 h of data. It should be noted that the current swallow detection algorithms are performed offline on computers. Since the detection algorithms are implemented in Java, they can be easily ported to smartphones. Performance on smartphones in terms of speed and power are not covered in this paper. The advantage of using an embedded wireless link is that the developed swallow sensor can be networked with other physiological [20] and physical activity sensors [3] to develop a networked sensing/detection system to provide a complete monitoring instrumentation package in the future.

A piezoelectric-based commercial belt (1132 Pneumotrace II, UFI Instruments, Morro Bay, CA) is adopted for breathing signal collection. Compared to a conductive rubber belt, it has higher linearity and smoother transient responses. Unlike with RIP, piezoelectric sensors do not need a loop connection or power sources. This makes them more suitable for embedding into clothes for better convenience and comfort.

### Swallowing and Apnea

**Fig. 2:**
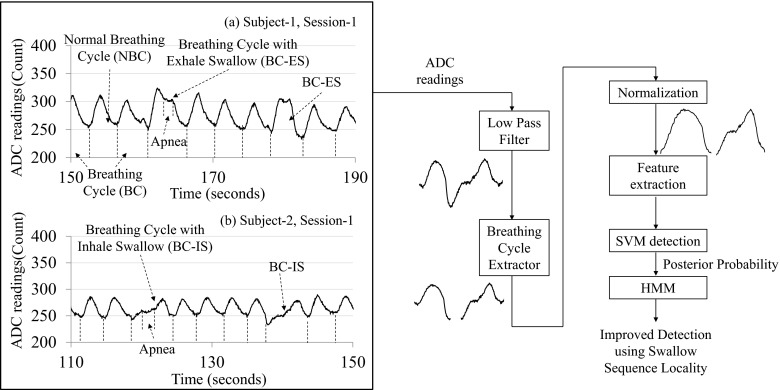
Respiratory signal with swallow signature (*left*) and swallow detection modules (*right*)

Figure 2 (left side) shows a number of experimentally obtained breathing signal segments for different human subjects. The ADC readings in the figure are directly proportional to the elongation and contraction of the piezoelectric sensing belt. The rising edges correspond to inhalations and the falling edges correspond to exhalations. As shown in the figure, a BC can be either normal (i.e., normal BC) or elongated due to swallow-triggered apnea. A cycle that is elongated due to an apnea at the beginning of an exhale (see top figure on the left in Fig. 2 for subject 1, session 1) is denoted as a BC with exhale swallow. For a second subject, the bottom figure on the left in Fig. 2 shows swallows (i.e., apnea) during the inhale process, which are denoted as BCs with inhale swallow.

### Detection Scheme

Figure 2 depicts the logic for classifying BCs on the right. Before sending the data to the ADC, an anti-aliasing analog low-pass filter circuit with a cutoff frequency of 30 Hz is applied. The signal is then sampled by the ADC at 100 Hz and fed into a software-based low-pass filter to remove quantization noise caused during the A–D conversion. Because the power spectrum of breathing signals is mainly below 2.5 Hz, 100 Hz is a sufficient sampling rate. The next step is to run the filtered data stream through a peak and valley detection software module to extract individual BCs. In order to perform peak and valley detection, the data stream is first divided into 10-s windows with 30% overlap, and then a threshold-based algorithm [21] is used. The threshold is set to 0.3(max_*d*(*m*)∈*C*_ *d*(*m*) − min_*d*(*n*)∈*C*_ *d*(*n*)), where *C* is a set that includes all the data points in the 10-s window, and *d*(*m*) and *d*(*n*) are the *m*th and *n*th sample points in the 10-s window. Data from one session of the experiment in the Sect. 3 are used for testing the performance of the proposed peak and valley detection algorithm. Table 1 shows the performance of BC extraction using peak and valley detection with various threshold values. Based on the data collected during one experimental session (see the Sect. 3), an optimal threshold of 0.3 was chosen. A threshold of 0.3(max_*d*(*m*)∈*C*_ *d*(*m*) − min_*d*(*n*)∈*C*_ *d*(*n*)) provides an accuracy of 99%.

**Table 1:** Accuracy of breathing cycle extraction using peak and valley detection

Threshold	Accuracy (%)
0.2(max_*d*(*m*)∈*C*_ *d*(*m*) − min_*d*(*n*)∈*C*_ *d*(*n*))	84
0.3(max_*d*(*m*)∈*C*_ *d*(*m*) − min_*d*(*n*)∈*C*_ *d*(*n*))	99
0.4(max_*d*(*m*)∈*C*_ *d*(*m*) − min_*d*(*n*)∈*C*_ *d*(*n*))	90

After individual BCs have been extracted, they are normalized in both time and amplitude dimensions. Each cycle is normalized to be between 0 and 100 vertically, and interpolated to 128 sample points in time. Considering an average length of a BC of 3.77 s in our experiments, the normalized sampling rate after interpolation is mapped to 34 Hz. Although different cycles may originally have different time and amplitude ranges (person-to-person or cycle-to-cycle for a given person), the normalization process removes such variance in duration and amplitude, thus making the cycles more suitable for the apnea detection process.

The feature extraction module takes BCs before and after normalization and extracts the following features: (1) BC length, (2) inhalation duration and depth, (3) exhalation duration and depth of BCs before normalization, (4) ±10 crossing counts, and (5) first five fast Fourier transform (FFT) coefficients of normalized BCs. Details of the extracted features are demonstrated in a later section. The features are then fed into the SVM detection module with posterior probability outputs, which are illustrated in more detail in a later section. At this stage, a posterior probability indicates the SVM-detected probability of a given BC to be of the type normal breathing or breathing with swallows. Information about swallow sequence locality is not utilized at this stage. Finally, the HMM is applied to the posterior probability outputs of the SVM module to improve detection performance by leveraging a priori knowledge about swallow sequence locality.

### Two-Tier Swallow Detection

In our previous work [22, 23], SVM was shown to be the best classifier for liquid swallow detection. As in traditional usage of SVM [24], the classification output for each BC was a class label (i.e., normal breathing or breathing with swallows). After analyzing the classification errors, it was realized that many of the errors can be corrected by applying known locality information in human swallow sequences. For example, people rarely swallow in many consecutive BCs. Thus, whenever the classification output shows many consecutive BCs, errors can be anticipated and the misclassified instances can be identified/removed by applying higher-level techniques such as the HMM. As in many similar applications, HMM is effective in leveraging temporal context information to improve accuracy. This motivates the proposed two-tier detection using SVM and HMM.

### SVM-Based Swallow Detection with Posterior Probability

Consider the following training set of size *T:*

$$\left( {x_{1} ,\;y_{1} } \right),\;\left( {x_{2} ,\;y_{2} } \right),\;\left( {x_{3} ,\;y_{3} } \right), \ldots ,\left( {x_{T} ,\;y_{T} } \right).$$

In each training instance (*x*_*i*_, *y*_*i*_), *x*_*i* ∈_ *R* ^*n*^ represents a set if *n* input features, and *y*_*i*_ is the corresponding class label. For a binary class system in our case, *y*_*i*_ can be defined as:

$$\left\{ {\begin{array}{ll} {y_{i} = 1} & {if\;x_{i} \in Breathing\;cycle\;with\;swallows}, \\ {y_{i} = {-} 1} & {if\;x_{i} \in Normal\;breathing}, \\ \end{array} ,\quad i = 1,\;2, \ldots ,T} \right..$$

A traditional SVM decision function can be derived as [25]:

1 $$D(x) = \sum\limits_{k = 1}^{T} {y_{k} a_{k} K\left( {x_{k} ,\;x} \right) + b} ,$$

where *a*_*k*_ and *b* are trained using the training data set, *T* is the number of training instances, and *K*(*x*_*k*_, *x*) is the kernel function of the SVM. Classification for a test feature set *x*_*j*_ using the decision function can conducted as follows:

$$\left\{ {\begin{array}{ll} {x_{j} \in Breathing\;cycle\;with\;swallows}, & {if\;D(x_{j} ) > 0}, \\ {x_{j} \in Normal\;breathing}, & {otherwise}. \\ \end{array} } \right.$$

The distance between *x*_*j*_ and the decision boundary (i.e., the boundary that separates a BC with swallows and normal breathing) with the maximum margin can be expressed as *D*(*x*_*j*_)/*C* [25], where *C* is a positive constant depending on *a*_*k*_ (*k* = 1, 2,…,*T*), training feature set *x*_*k*_ (*k* = 1, 2,…,*T*), and the kernel function. Therefore, |*D*(*x*_*j*_)| is positively correlated to the confidence of correct detection; that is, the closer it is to the decision boundary, the less confidence in correct classification.

In order for the HMM to be able to process the SVM output, the latter needs to be in the form of posterior probability [25] as opposed to the class labels used by traditional SVM models [24], as described above. An appropriately designed SVM [25] can indicate the probability that a given input feature set corresponds to a specific class. This probability is referred to as the posterior probability for that class. In what follows, we describe the mechanisms for computing such probabilities, which are the input for the swallow-sequence-based HMM.

Posterior probability for class_i_ is formally defined as *prob*(*class*_*i*_|*input features*) = *prob*(±1|*x*_*i*_). This indicates the probability that a given input feature set *x*_*i*_ corresponds to a BC with swallows or a normal BC. It follows that *prob*(1|*x*_*i*_) + *prob*(−1|*x*_*i*_) = 1. We use the following method for computing posterior probability using the SVM decision function *D*(*x*_*j*_), as proposed by Wahba [26]:

2 $$prob\left( {class_{i} |input\;features} \right) = prob(y = 1|x) = \frac{1}{1 + \exp (A\;*\;D(x) + B)},$$

where *A* and *B* are constants and estimated by minimizing the negative log likelihood of the training data set using regression methods.

### Hidden Markov Model with Swallow Sequence Locality

The key concept of HMM in swallow detection is as follows. A sequence of BCs is represented by a discrete time Markov chain consisting of two states (i.e., normal BCs and BCs with swallows) that are hidden from an observer, meaning that the observer cannot directly determine which state the system is in at any given point of time. However, the posterior probability out of the SVM, which indicates the likelihood of the system being in any state, is visible to the observer. The idea of the HMM formulation is that if the locality in swallow sequence dynamics and the mapping between the system’s state and posterior probability observation are known (or measurable) to the HMM model, then by observing the posterior probability out of the SVM, the current state in the Markov chain can be estimated.

#### Hidden State Space

**Fig. 3:**
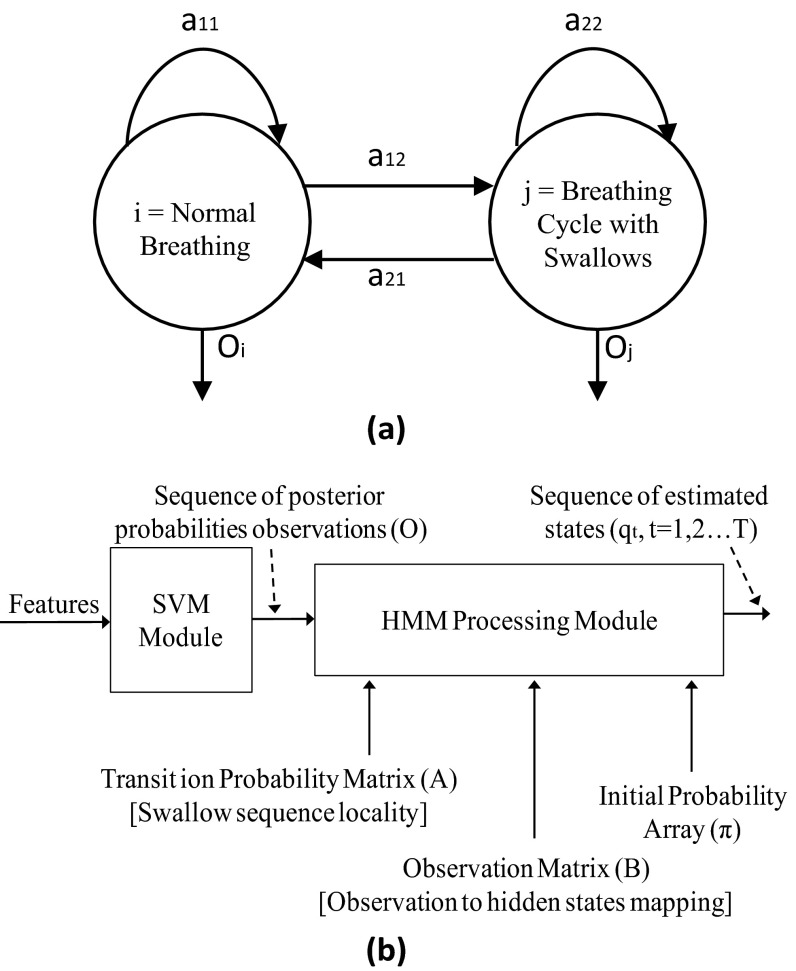
Hidden breathing state machine

As shown in Fig. 3, a BC sequence can be modeled as a hidden state machine with two hidden states, namely normal breathing and BC with swallows. The states are hidden because they are not deterministically known from posterior probabilities computed out of the SVM processing.

#### Transition Probability Matrix

It is defined as *A* = {*a*_*ij*_}, where *a*_*ij*_ represents the probability of transitioning from state *S*_*i*_ to state *S*_*j*_.

$$a_{ij} = prob\left( {q_{k} = S_{j} |q_{k - 1} = S_{i} } \right).$$

It is assumed that *q*_*k*_ depends only on *q*_*k*−1_, which means:

$$prob\left( {q_{k} |q_{k - 1} } \right) = prob\left( {q_{k} |q_{k - 1} ,\;q_{k - 2} , \ldots ,q_{1} } \right).$$

*A* is a 2 × 2 matrix for two BC types in our case. The transition probability matrix is constructed from the true swallow sequence detected by a video camera and the push button in our experimental apparatus, as shown in Fig. 1. The probabilities in this matrix represent the swallow sequence locality information, which is leveraged by the HMM processing.

#### Observation Matrix

Although the states are considered hidden, the SVM-computed posterior probability at each state can be considered as an observable parameter for HMM modeling purposes. For a given state_i_, the posterior probability *prob*(*y*_*i*_ = 1|*x*_*i*_), generated by the SVM detector, is utilized for constructing an observation bitmap *O*_*i*_ in the following manner.

The posterior probability from the SVM in the range [0, 1] is divided into N equal windows (we used N = 10). Each window is represented as a bit in the N-bit long bitmap *O*_*i*_. The bit corresponding to the window in which the posterior probability *prob*(*y*_*i*_ = 1|*x*_*i*_) falls on is set to 1, and all other bits in *O*_*i*_ are set to 0. For example, with N = 10 and *prob*(*y*_*i*_ = 1|*x*_*i*_) = 0.71, the observation bitmap for state_i_ will be *O*_*i*_ = {0, 0, 0, 0, 0, 0, 0, 1, 0, 0}, with the eighth big set to 1 since 0.7 < *prob*(*y*_*i*_ = 1|*x*_*i*_) = 0.71 ≤ 0.8.

Now let *b*_*jm*_ be the probability that if an observation bitmap’s *m*th bit is 1 (i.e., all other bits are 0s), then the system is in hidden state j. Formally stated:

$$b_{jm} = prob\left( {O = \left\{ {bit_{1} = 0, \ldots ,bit_{m} = 1, \ldots } \right\}|State = S_{j} } \right).$$

An observation matrix of size *M* × *N* (*M* number of states, *N* number of bits in the observation bitmap) is constructed as *B* = {*b*_*jm*_}. In this case, a 2 × 10 matrix is constructed by combining the true swallow events detected by a video camera and the push button, and the SVM outputs *prob*(*y*_*i*_ = 1|*x*_*i*_) after processing the chest belt sensor data. This observation matrix, together with the transition probabilities and the following initial probability array, is used for HMM processing.

#### Initial Probability Array

The initial probability array is represented by a vector *π* = [*π*_*i*_] of length *M* (i.e., 2), in which:

$$\pi_{i} = prob\left( {q_{0} = S_{i} } \right)\quad 1 \le i \le M.$$

*π*_*i*_ indicates the probability that the initial state of the hidden state machine is *S*_*i*_. Therefore, by definition:

$$\sum\limits_{i = 1}^{M} {\pi_{i} = 1} .$$

This array is formed using true swallow data gathered by the experimental system, as described in the Sect. 2. The swallow system as modeled by HMM can be expressed as a tuple: *λ* = (*A*, *B*, *π*), where *A*, *B*, and represent the hidden state transition matrix (i.e., known swallow sequence locality), observation locality, and initial condition of the state machine, respectively.

## Results and Discussion

### Experimental Design

**Fig. 4:**
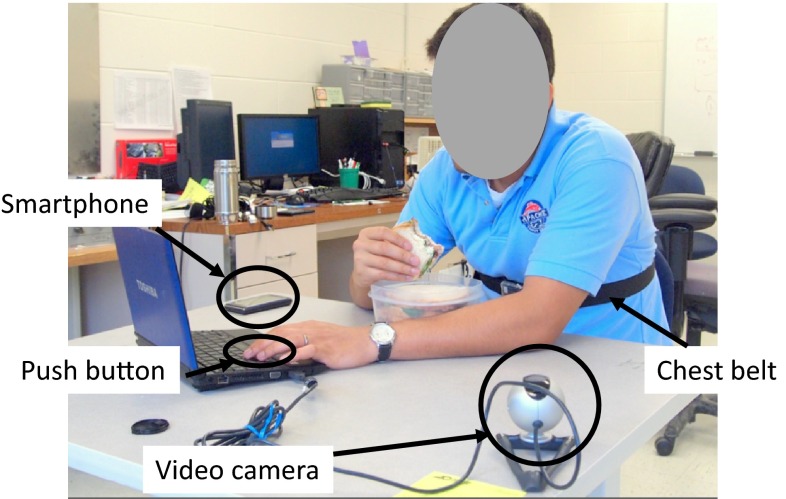
Experimental setup

During the experiment, a subject was instructed to press a button whenever they swallowed, and the smartphone shown in Fig. 1 was used to record the breathing signals sensed by the wireless chest belt. A video camera was connected to a computer to record the movement of the mouth and the laryngopharynx during the experiment for validation purposes. The computer, smartphone, and button recorder were synchronized before each experiment session. The experiment setup is shown in Fig. 4.

Experiments were carried out on six subjects (two female and four male) without any known swallow abnormalities. Each subject was asked to conduct three experiment sessions. During an experiment session, the subject was asked to wear the instrumented chest belt and have their lunch at their own pace. The lunch type was chosen by individual subjects based on their dietary preferences. Lunches included diverse food types, including rice, bread, salad, and cooked vegetarian and non-vegetarian items. Note that the subjects were allowed to drink during the experiments. However, since this study focused on only solid intake, BCs during drinking were first identified from the video recording, and then removed during data processing. Each experiment lasted around 10–15 min with 200–300 BCs depending on the eating speed and the amount of food, and thus for each subject 600–900 BCs of data were collected. Leave-one-out validation, i.e., data from each subject was used for testing while those of others were used for training, was applied in the data processing to avoid over-fitting. For each training and testing iteration, 3000–4500 BCs from five subjects were used for training, and 600–900 BCs of the last subject were used as the testing data set. This process was repeated using each subject’s data as the testing data set. The average accuracy of using each subject’s data as the testing data set gives us a good performance estimation of the system when used on future subjects whose data have not been used for training the model.

It should be noted that the subjects were restricted from sleeping, running, walking, or moving exaggeratedly during the experiments. As part of a complete monitoring instrumentation package with other physiological and physical activity sensors (see Fig. 1), the food intake detection algorithm was suspended during sleeping, running, and other vigorous activities. This is reasonable since it is not very common for people to eat during such activities.

### Performance Indices

To evaluate the detection performance (i.e., both SVM only and SVM combined with HMM), we adopted the metrics precision and recall, which are commonly used [5, 6] in biomedical signal processing and information retrieval. Precision and recall are defined as:

$$Precision = \frac{TP}{TP + FP} = \frac{Recognized\;swallows}{Retrieved\;swallows},$$

$$Recall = \frac{TP}{P} = \frac{Recognized\;swallows}{Relevant\;swallows}.$$

In this definition, recognized swallows (i.e., true positives, TPs) indicate the number of swallow events that were correctly detected. Retrieved swallows correspond to the number of detected swallows, including both TPs and incorrectly detected swallows (i.e., false positives, FPs). Relevant swallows (i.e., positive, P) refer to the number of actual swallow events annotated from video, observations reflecting the ground truth.

### Results and Discussion

**Fig. 5:**
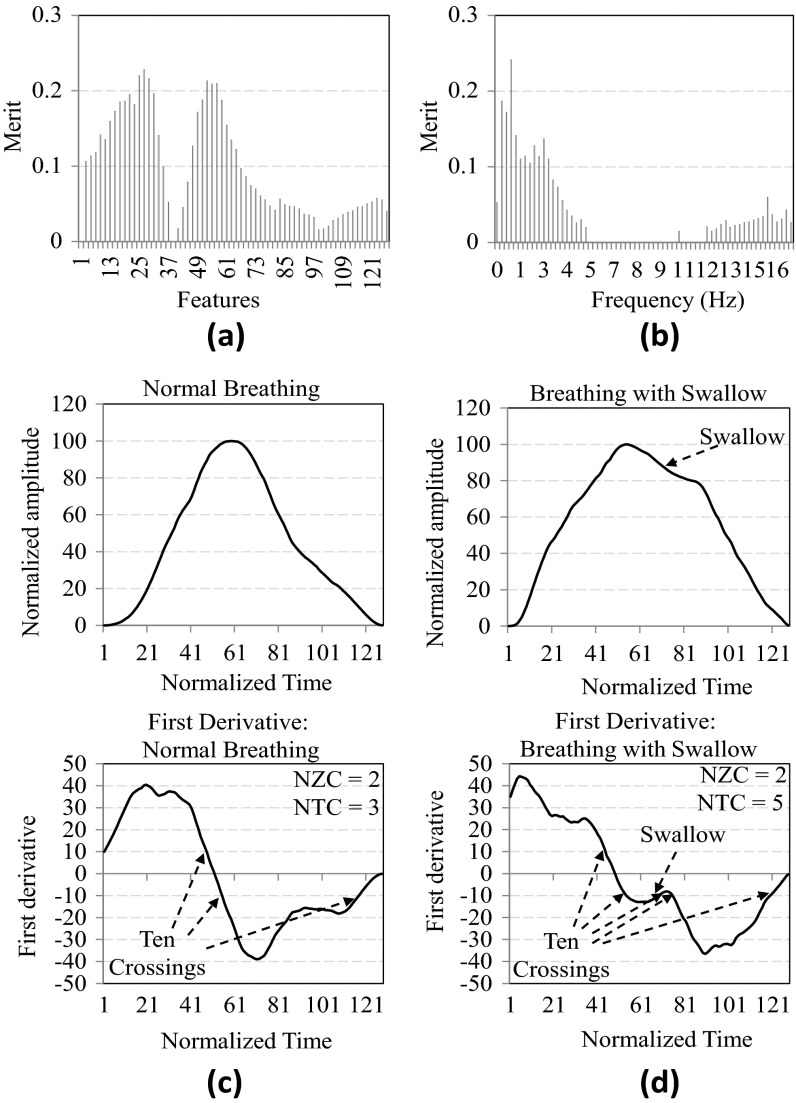
Feature discriminative property and ±10 crossings as classification feature

Both time- and frequency-domain features can be used for detecting liquid swallows. The discriminative power of these feature types, however, can be different. Figure 5 shows the discrimination power of time- (Fig. 5a) and frequency-domain (Fig. 5b) features in solid swallow detection using the SVM classifier. The merit of a feature in Fig. 5a, b refers to information gain [27], which is defined as the reduction in classification entropy [i.e., *H*(*)] with additional information provided by the corresponding feature about the target classes. Assuming *A* as the feature set and *C* as the set of classes, i.e., normal BC and BC with swallows, the following two equations indicate class entropies before and after the use of the feature, where *a* represents a feature in *A* and *c* represents a class in *C*:

$$H(C) = {-} \sum\limits_{c \in C} {p(c)\log_{2} p(c)},$$

$$H(C|A) = {-} \sum\limits_{a \in A} {p(a)\sum\limits_{c \in C} {p(c|a)\log_{2} p(c|a)} }.$$

A feature with higher merit indicates lower class entropy when this feature is adopted. Merit can be also used when feature reduction is needed in the presence of limited computational and storage resources.

Figure 5a, b depict the merit of time- and frequency-domain features, and Fig. 5c, d show the use of ±10 crossing features. For time-domain features, as shown in Fig. 5a, where 128 sample points in normalized BCs are used as features, sample points near the 27th and 53rd sample points are the most important for classification. For frequency-domain features, as shown in Fig. 5b, where the first 64 FFT coefficients are used as features, lower-frequency components have the most discriminative power. It was also found that the discriminative power distribution of frequency-domain features is more consistent across subjects than time-domain features, which is why frequency-domain features were used in a previous study [22].

The second set of SVM classification features is derived from the first derivative of the breathing signal. As shown in the Sect. 2, it was found that the swallows generally create more fluctuations in breathing signals compared to those created by normal BCs. To capture such fluctuations, an additional classification feature was derived from the first derivatives of the breathing signal. More specifically, the number of ±10 crossings (NTCs) is used as a feature, which is defined as the number of points in the breathing signal at which the first derivative of the signal is exactly +10 or −10. Compared to the number of zero crossings (NZC), NTC can better capture swallow signatures.

Figure 5c, d show an example comparison between a representative normal BC and a representative BC with swallows and their corresponding NZC and NTC of the first derivatives. For both types of BC, NZC is 2, but NTC is 3 for normal breathing and 5 for breathing with swallows. The additional 2 NTCs (i.e., −10 crossings) are contributed by the swallow event. Differences in NTCs were consistently observed between BCs with and without swallows, thus indicating the usefulness of the NTC of the first derivative as a useful classification feature for the SVM engine.

The third set of features is derived from the duration and amplitude of the BCs before normalization. In summary, the SVM features used in this paper are: first five Fourier transform coefficients, NTC, inhalation duration, exhalation duration, total BC duration, inhalation amplitude, and exhalation amplitude.

It should be noted that we used leave-one-out validation in this paper, and the optimized feature set for one subject may be different from that for another subject. In order to be generalizable, we used the same feature set for all subjects.

### Swallow Detection with SVM

The features mentioned above were fed into the posterior probability SVM classifier described in the Sect. 2. The classifier was trained and validated using data collected through experiments outlined in Fig. 4. We used a leave-one-out validation approach, in which a subject’s data are excluded in the training set if their data are used as the test set.

**Fig. 6:**
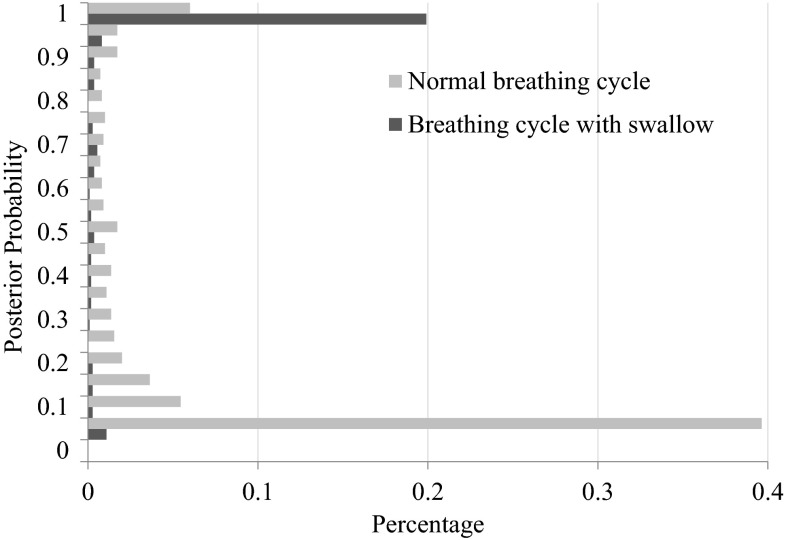
Distribution of posterior probabilities with and without swallows

Figure 6 reports the distribution of the SVM-produced posterior probabilities (i.e., the probability of a BC being a BC with swallows). The distribution was plotted from all BCs obtained during the experiment. The posterior probability is first quantized as follows: 0.1 for posterior probability falling in [0–0.1], 0.2 for (0.1–0.2],…,1 for (0.9–1.0]. The percentage of BCs in each region is plotted as bars in Fig. 6. In the absence of classification errors, there would have been only one bar at probability 1 for the cycles with swallows. Similarly, there would have been only one bar at probability 0 for the cycles without swallows. In Fig. 6, it can be observed that in spite of some classification errors (i.e., indicated by the scattered bars over the probability axis), the SVM is able to separate the two cycle types fairly distinctly. Such errors are often caused by swallow signatures that are too short (in time) to be captured by the specified features, and by BC modulation by adjacent swallows [13].

By applying a probability threshold Pth to the SVM-produced posterior probabilities, it is possible to classify each cycle as a normal or swallow-containing BC in the following manner.

$$\left\{ {\begin{array}{ll} {normal\;breathing\;cycle}, & {if\; prob(1|x_{i} ) < Pth}, \\ {breathing\;cycle\;with swallow}, & {otherwise}. \\ \end{array} } \right.$$

**Fig. 7:**
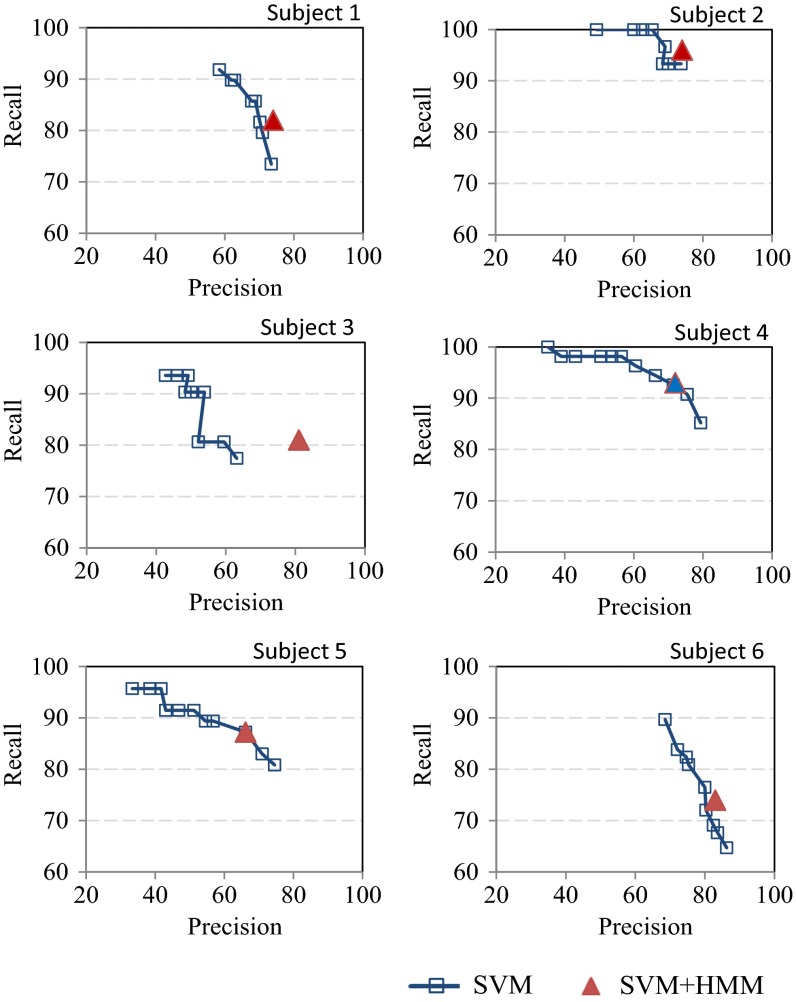
Comparison between SVM-only and two-tier SVM + HMM mechanism

Figure 7 shows the SVM-only classification accuracy (i.e., precision and recall) for threshold Pth values of 0.1–0.9. Each point on the SVM curve for a given subject corresponds to a precision and recall pair for a given threshold value. When the threshold is increased, precision increases, whereas recall decreases, indicating fewer FPs and TPs. Since the breathing signals during lunch are imbalanced (i.e., there are many more normal BCs than BCs with swallows), a higher threshold gives more preference over normal BCs and therefore reduces the number of FPs, thus increasing precision and decreasing recall. The reverse effect was observed when the detection threshold Pth was decreased. The SVM-only performance lines in Fig. 7 provide a means for choosing an appropriate Pth for a required balance between precision and recall. Table 2 presents the precision and recall performance for all six subjects obtained with a threshold of 0.5 for swallow classification.

**Table 2:** Performance summary of SVM and SVM + HMM schemes

	SVM		SVM + HMM	
	Precision (%)	Recall (%)	Precision (%)	Recall (%)
Subject 1	68	86	74	82
Subject 2	67	100	74	96
Subject 3	49	90	81	81
Subject 4	54	98	72	93
Subject 5	45	91	66	87
Subject 6	80	80	83	74
Average	61	91	75	86

### Improved Detection Using HMM

HMM processing, as outlined in the Sect. 2, was applied to the posterior probabilities out of the SVM to improve detection performance. Such improvements are accomplished by correcting some of the SVM errors by leveraging known locality information in human swallow sequences. As described in the Sect. 2, for each subject, the A (2 × 2) matrix is derived by computing the transition probability between the two states (i.e., normal BCs and BCs with swallows) using the ground truth indicated by the pushed button results. The B (2 × 10) matrix is calculated from the posterior probability from the SVM module and the given ground truth. Each element thus represents the probability that a 1 appears in one of the N positions, given the state (i.e., normal BC or BC with swallows), as illustrated in the Sect. 2. As an example, for subject 1, the *A* matrix was found to be:

$$A = \left[ {\begin{array}{cc} {0.76} & {0.24} \\ {0.96} & {0.04} \\ \end{array} } \right]$$

and the *B* matrix was found to be:

$$A = \left[ {\begin{array}{cccccccccc} {0.84} & {0.03} & {0.01} & 0 & {0.03} & {0.01} & {0.01} & {0.01} & {0.01} & {0.06} \\ {0.08} & {0.02} & 0 & 0 & {0.04} & 0 & {0.04} & {0.02} & {0.06} & {0.74} \\ \end{array} } \right].$$

The swallow detection performance of the SVM + HMM approach is presented in Fig. 7. For each subject, there is one point that indicates the corresponding precision and recall performance. Observe that for subjects 1, 2, 3, and 6, the SVM + HMM point is situated higher and on the right in comparison to the line for the SVM-only approach. This indicates better performance of SVM + HMM compared to the SVM-only approach with all possible posterior probability thresholds. For the remaining two subjects (i.e., 4 and 5), the SVM + HMM performance point is on the SVM-only line, indicating that with certain posterior probability thresholds, SVM-only can perform as well as the SVM + HMM approach. The variation of performance across subjects results from the different way people eat solid food. For example, some people eat faster, and thus have shorter intervals between consecutive swallows, larger bolus sizes, or both.

Table 2 compares the performance of the SVM-only solution using a threshold of 0.5 with SVM + HMM. It demonstrates that SVM + HMM consistently outperforms the SVM-only solution when the optimum threshold of posterior probability is unknown.

These results validate the overall usefulness of the proposed HMM processing that leverages known swallow sequence locality information for removing certain classification errors that are introduced by the SVM-only approach.

### Performance of Existing Approaches

Passler and Fischer [6] achieved 91.3% precision and 81.8% recall with an in-ear microphone, with the subjects restricted from talking, and ambient noise minimized to reduce artifacts. In another study [28], inertial sensors were used to track the movements of the arms and trunk. An in-ear microphone was used to record the food breakdown sound, and sEMG electrodes and a stethoscope microphone were deployed to detect swallowing activities. A precision of 20% and a recall of 68% were achieved. Makeyev and colleagues [10] used a throat microphone to detect swallowing; the average accuracy was 66.7% for the inter-subject model (cross-validation).

In this paper, as shown in Table 2, the SVM + HMM had a precision of 75% and a recall of 86%, which are comparable to, or better than, the reported performance in the literature under comparable ambient and artifact scenarios.

A detailed analysis of mistakenly detected BCs revealed that the main cause for loss of accuracy is irregular breathing during food intake caused by feeding and chewing. More specifically, people tend to feed themselves during inhalation and might subconsciously hold their breath for a short period of time. During chewing, people sometimes unnoticeably swallow saliva with a very small amount of food.

## Conclusion

This paper proposed a wearable wireless solid food intake monitoring system. A processing mechanism based on SVM and HMM, which analyzes collected breathing signals and known swallow sequence locality information, was also proposed. The system and processing mechanism were experimentally proven to be effective for solid food intake detection. Ongoing work on this topic includes the following. (1) Development of a generalized unsupervised swallow detection mechanism for both solid and liquid swallows. It was found that people are more likely to eat solid food continuously over a period a time, while drinking usually happens with one or two gulps at a time during a very short period. During drinking, the feeding action usually coincides with the swallow, while for eating, people need to chew the food into a bolus before swallowing. (2) Development of a detection and filtering mechanism for artifacts introduced by movement and speech. During talking, the vocal fold modulates the expiration air flow to produce sound, which is different from apnea caused by swallows. It is not very common that people eat while walking, during which the breathing signals are modulated by a periodic signal caused by steps. It is feasible to extract step signals from breathing signals using accelerometer data available on smartphones. (3) Implementation of a real-time swallow detection system that can be used by health researchers for retrieving dietary information from a targeted population. (4) System feasibility testing for long experimental duration (e.g., days). (5) Addition of a sensing modality into the belt, such as an accelerometer to detect trunk movement, and integration of the food intake monitoring system with other physiological [20] and physical activity sensors [3] to develop a networked sensing/detection system to provide a complete instrumentation package for calorie management.
